# Preservation of iridescent colours in *Phorinia* Robineau-Desvoidy, 1830 (Diptera: Tachinidae)

**DOI:** 10.3897/BDJ.4.e5407

**Published:** 2016-01-07

**Authors:** Yves Braet, Stephen Downes, Priscilla Simonis

**Affiliations:** ‡ Institut Royal des Sciences Naturelles de Belgique, Rue Vautier 29, Brussels, Belgium; § Eades Farmhouse, Church Road, Theberton, Suffolk, United Kingdom; | Photonic of living Organisms group, Research Center in Physics of Matter and Radiation (PMR), University of Namur (UNamur), 61 rue de Bruxelles, B-5000 Namur, Belgium

**Keywords:** Entomology, Structural colour, Photonic crystal, Pigments, Iridescence

## Abstract

**Background:**

Iridescent blue-green colours are exhibited by various organisms including several taxa in the Tachinidae (Diptera) with notable examples within the Afrotropical members of the genus *Phorinia* Robineau-Desvoidy, 1830. The vivid colouration observed in life quickly fades to a dull golden-yellow when a specimen is dried. Although well known, no published explanation has been given for this phenomenon.

**New information:**

We illustrate the mechanism associated with this colour change. We also test and propose technical alternatives to retain the living colours in dried specimens.

## Introduction

The Tachinidae is one of the largest Diptera family with approximately 8,500 species in more than 1,500 genera worldwide distributed among the 4 subfamilies (Crosskey 1973, Crosskey 1976, Crosskey 1977, Crosskey 1980, Pape et al. 2011, Cerretti et al. 2012, O'Hara 2013, O'Hara 2014). The larval stages of Tachinidae are endoparasitoids of arthropod groups (Crosskey 1973, Crosskey 1976, Belshaw 1994, Stireman et al. 2006), exhibiting various reproductive strategies from indirect oviposition to direct internal or external oviposition with several cases of ovolarviparity (Stireman et al. 2006). The lack of detailed information on host associations across the whole family, together with the largely unresolved phylogenetic relationships of the Tachinidae, makes it difficult to understand evolutionary patterns of host use (Stireman et al. 2006, Tachi and Shima 2009). The recent work of Cerretti et al. 2014a recovers only the subfamilies, Exoristinae and Phasiinae as monophyletic.

The genus *Phorinia* has traditionally been placed in the oviparous tribe Exoristini of the subfamily Exoristinae based on morphological autapomorphies (strongly reduced female eighth abdominal sternum; facial ridge with strong setae; occiput without rows of black setae behind postocular setae; R4+5 setulose dorsally from base nearly to crossvein rm; apical scutellar setae erect)(Tachi and Shima 2006). Even if this genus is recovered as monophyletic and sister-group of *Ctenophorinia* Mesnil, 1963 (Tachi and Shima 2006), its broader phylogenetic relationships are unclear. Indeed, Tachi (2011), Tachi (2013) and Tachi and Shima 2009 suggested that these genera should be excluded from the tribe Exoristini based on molecular and morphological data. However, the monophyly of Exoristini including these two genera is not rejected by recent analyses (Tachi 2013, Cerretti et al. 2014a). *Phorinia* contains sixteen species from the Palearctic and Oriental regions (Crosskey 1976, Crosskey 1977, Crosskey 1980, Tachi and Shima 2006). Hosts of *Phorinia* are known for a few West Palaearctic and Afrotropical species, with records as parasitoids of lepidopteran larvae (families Geometridae, Epiplemidae, and Noctuidae) (Herting 1960, Lehrer and Plugarj 1966, Mesnil 1971, Tschorsnig and Herting 1994). Scant attention has been given to the seven described species from the Afrotropical region which may form a taxonomically separate group of species (Mesnil 1971, Tachi and Shima 2006). Afrotropical *Phorinia* species have an obvious character in the presence of large tomentose ("pruinosité", in French) areas exhibiting vivid blue-green colours mainly on the thorax, but also on dorsal and lateral parts of head and abdomen. The tomentose areas are composed of dense microscopic hairs. A dense microscopic tomentosity without iridescence is commonly observed in many dipteran families, and sometimes it is highly reflective at certain angles and provides a contrasting pattern to the underlying dark cuticle. Within the subfamily Exoristinae (Diptera: Tachinidae), other genera also exhibit iridescent tomentum, including the East Palearctic *Ctenophorinia* Mesnil, 1963, the Neotropical *Chrysoexorista* Townsend, 1915, *Eulobomyia* Woodley & Arnaud, 2008 and the Afrotropical *Blepharella* Macquart, 1851 (Ziegler and Shima 1996, Woodley and Arnaud 2008a, Woodley and Arnaud 2008b, Raper 2010). Unlike the scales of Lepidoptera, this iridescence is rapidly lost on drying, fading to a dull golden yellow (straw) colour. Notably, immediate conservation in 70% alcohol, via a malaise trap for example, retains the vivid colouration.

In nature, there are two major mechanisms to produce colouration in organisms: by light absorbing pigments and by structural interference (Stavenga 2014). These two mechanisms may be utilised alone or together to obtain visual effects. Pigmentation is usually effected by a small class of organic compounds. For example, carotenoids (sourced from plants) absorb shorter wavelengths of light and allow longer wavelengths to be transmitted or reflected depending on the composition of the surrounding material. This results in brown, red, orange or yellow colouration (Fox 1976, Shamim et al. 2014). In comparison, melanin pigments have high absorbance in all visible wavelengths, resulting in brown to black colours (Fox 1976). Some pigments or dyes are highly specific to a group of taxa like papiliochromes in Papilionidae, pterins in Pieridae, ommochromes in Nymphalidae and fluorescent pigments in Apidae; which are produced through specialized biological pathways (Nemésio 2005, Shamim et al. 2014).

Structural colours are produced by the physical interaction of light with the nanometre-scale variation in the integumentary tissues of animals and plants. The standard mechanisms responsible for producing structural colour have been defined, studied and reviewed in several works over the last 40 years (e.g. Vigneron et al. 2005, Kinoshita and Yoshioka 2005, Simonis and Berthier 2012, Vignolini et al. 2012, Sun et al. 2013, Simonis et al. 2013). These colours are produced when light interacts at boundaries of media with different refractive indices, where, depending on the dimensions of the media, some wavelengths constructively interfere to produce brilliant colours, while the remaining wavelengths destructively interfere (Prum 2006, Kinoshita et al. 2008, Shevtsova et al. 2011). This interference is able to produce iridescent colours which are highly directional, changing in appearance with the observer’s relative position. Such colouration can be found in plants (Gould and Lee 1995, Lee 2007), but is more widespread in animals, from birds’ feathers to cephalopod iridophores and arthropod exoskeletons. Some of the most well-known examples are found in the cuticles of Coleoptera and the wing scales of Lepidoptera (e.g. Cooper and Hanlon 1986, Mathger et al. 2009, Vigneron et al. 2009, Shevtsova et al. 2011, Saranathan et al. 2012, Vignolini et al. 2012, Colomer et al. 2012). An important feature of structural colours is that they allow organisms to produce colours that cannot easily be obtained with pigments. Blue colours, often with metallic reflections (Kemp 2007, Lim and Li 2013), are usually structural, as blue pigments are rare in nature. Only a few invertebrate species are known to use blue pigments for colouration (Simonis and Berthier 2012, Simonis et al. 2013).

Colours and visual effects are often used to enhance cryptic or aposematic appearance and are also used in mate selection (McGraw et al. 2002, Kuitunen and Gorb 2011, Meadows et al. 2012, Kemp et al. 2014). Some organisms even can alter their appearance dynamically in response to abiotic or biotic pressures (Shand 1988, Fitzstephens and Getty 2000). Insects have a rich diversity of utilising combinations of pigmentsand structural colours. The Lepidoptera, Coleoptera and Hymenoptera contain many such examples: anthocyanins help in mate selection in the butterfly, *Polyommatus icarus* (Rottemburg, 1775)(Lepidoptera: Lycaenidae) but in combination with melanin act as a warning colouration in *Parasemia plantaginis* (Linnaeus, 1758)(Lepidoptera: Arctiidae) larvae (Lindstedt et al. 2010); the diffuse green reflectance of the elytra in *Entimus imperialis* (Forster, 1771) (Coleoptera: Curculionidae) may play a role in intersexual recognition or/and provides cryptic camouflage when seen at long-distance (Wilts et al. 2012); in the Hymenoptera, some species of Orchid bees (Euglossinae) and Cuckoo wasps (Chrysididae) have a bright green, blue or purple iridescence produced by the multi-layered structure of their cuticles (Kroiss et al. 2009). In the Orthoptera, the grasshopper *Kosciuscola tristis* Sjöstedt, 1934 has a blue coloration which is thermochromic (the colour varying with temperature) (Umbers 2011). Within the Diptera, examples of bright metallic cuticles are found in several families, such as in the Stratiomyidae; *Ptilocera dentata* (Fabricius, 1805) and *Eudmeta marginata* (Fabricius, 1805), in some Calliphoridae (e.g., *Lucilia* spp.) and Dolichopodidae. These intricate natural colours have developed through evolution over millions of years. They often participate in other functions of an organism, giving rise to complex multi-scale and multifunctional structures (Berthier 2003). The study of structural colours is not only interesting for biologists but can also, by a reverse engineering process, be a strong source of inspiration to develop new materials for technical applications such as hygrometric detectors, microscopic films, thermal insulation and coloured fibres (Potyrailo et al. 2007, Rassart et al. 2008, Deparis et al. 2008, Vigneron et al. 2009, Vigneron et al. 2010, Simonis et al. 2013 etc.).

- We present a new hydrochromic (observed colour varies due to the absence or presence of water) structure found in *Phorinia* (Diptera: Tachinidae). We illustrate the macroscopic mechanism causing this colour change and also test and discuss curation methods to retain the bright colours in a dry collection. In addition, we raised some questions linked to the presence of this character.

## Material and methods

Several specimens of *Phorinia* were collected by Malaise traps, from 28.I - 30.II.2012, by the field mission of the “Insectes du Monde” NGO in the Dzangha-Ndoki National Park, Central African Republic (CAR) (http://www.insectesdumonde.org/spip.php?article51). Specimens were stored in 70% alcohol after the mission. Among the thousands of Diptera specimens collected, less than 50 were of Tachinidae with vivid blue-green colours. These belonged to the genera *Blepharella* (5 conspecific specimens) and *Phorinia* (45 specimens belonging to *P. veritus* Walker, 1849 and an undescribed species). Both genera are widespread in the Afrotropics.

Detailed images of the tomentum were produced using three specimens of *P. veritus*. The first specimen was dried normally in air at room temperature. This was dissected and coated with 2 nm of gold, with the remainder retained for colour photographs. The two remaining specimens were dehydrated in a graded ethanol series (50% to 100%) for 1 hour, followed by 12 hours in a 100% alcohol bath. These specimens were critical point dried (CPD) using a Balzers CPD 030 (Leica Microsystems 2014). One specimen was used for light microscope analysis photography. The other was coated with 2 nm of gold after mounting on aluminium stubs.

The coloured detail of scales at high magnifications has been realized with a Olympus DSX 500. Microphotographs of scales have been realized using a Phenom G2 Pro SEM apparatus (Phenom-World, Benelux Scientific, Belgium).

Four solvents (alcohol, acetone, formaldehyde dimethyl acetal, HDMS) and a mix of aliphatic solvents ("Detach tout"®) were tested for their capacities to retain the living colours and structure of scales in the tomentose areas. Small sections of head, thorax, and tergites from specimens in 70% ethanol were dehydrated in 2 x 2 baths of 90% and 100% ethanol for 1 hour each. The ethanol was then replaced by the solvent under test using a graded concentration series (from 10% to 100% of the solvent). At the end of the process, the samples were left to dry in open air at room temperature or in a heated glass vial (80°C). The colour was noted after 3 hours. Before testing a new solvent, the samples were rehydrated in 70% ethanol to restore the original blue-green colours. The same protocol was then reapplied with a subsequent solvent. We used a new specimen for the test if rehydration failed to restore the colouration.

## Results

As expected, the air drying resulted in a specimen where all the vivid blue-green tomentose areas (posterior genae, frons, mesosoma dorsally, anterior part of tergites 3, 4 and 5) transformed to a dull yellow colour (Suppl. material 1). The specimens dried with the critical point methods (CPD) successfully retained their vivid bluish-green colours over the majority of the tomentose areas. Detailed examination of these areas at high magnification (with both optical microscope and SEM) revealed that these are composed of a high density of specialized scales. By examination of the critical point dried specimens, we observed the scales vary somewhat in size (length: 6.2-19.1 µm; width: 2.5-6.2 µm) and taper apically producing a "rugby ball" type appearance (Fig. 1a, Fig. 2a, b). The scales’ colours are mostly the same vivid bluish-green as seen on the hydrated specimens. No clear iridescence was detected on these "rugby ball" scales (Fig. 1a).

With the air dried specimen, we found the scales flattened and weakly curved toward the cuticle (Fig. 1b, Fig. 2c, d). Their colours are clearly dull yellow on their edges, but their central part looks transparent with some random patches of colours varying from purple to blue (Fig. 1b). Under transmission light microscope, the flattened scales are fully pale yellow (data not shown).

After dehydration using Acetone or HDMS, we successfully achieved curation of the blue-green colour, though less vividly than in the hydrated stage for the scales on the piece of thorax, but the scales on the other body parts (head and tergites) still faded to a pale dull yellow or white colouration. With these two solvents, evaporation using heat accelerated the recovery of a dry specimen and improved colour retention on most parts of the specimen. SEM examination of these dehydrated scales on the thorax revealed that most remained inflated but less so than after CPD. The scales with dull yellow or white colouration are flattened similarly to the air dried samples.

The use of solvents other than Acetone or HDMS failed to preserve the original vivid colours. Samples retained their colours in the solvent but, on drying, became dull yellow at a rate depending on the solvent’s evaporation rate. Careful increase of the evaporation rate with an additional heat source did not improve the result. In all cases, immersing the dry sample back into the solvent or into water (for some hours) restored the original blue-green colour.

## Discussion

The curation of colours and body structures in insects has been a challenge for many years. Several protocols have been developed corresponding to both technical and chemical developments. These protocols start with two steps: the fixation of the sample (often with formaldehyde and/or glutaraldehyde, sometimes with subsequent use of osmium tetroxide) followed by dehydration using a graded series of an organic solvent (usually acetone or alcohol). The sample is then dried either by Critical Point Drying (CPD) or sublimation (Kan 1990). These two methods avoid producing the surface-tension and capillary effects which can adversely distort microscopic structure (Favret 2009). Alternative chemical approaches include using solvents with low surface tension such as hexamethyldisilazane (HMDS) (Nation 1983, Slížová et al. 2003, Buravkov et al. 2011) or Acetone (Van Noort 1996). Both methods result in greatly reduced deformation of the soft structures in less sclerotized insects. Another option would be to use sublimation such as with the Peldri II. But this is now difficult in practice since these fluorocarbon compounds are legally prohibited.

The results of our drying tests lead us to several observations and hypotheses. The internal structure of the scales is flexible though robust enough to not be altered by successive cycles of dehydration / hydration. The use of a solvent with a low surface tension, such as Acetone and HDMS improves the conservation of colour. Curation is improved with the use of a heat source to speed up the Acetone and HDMS evaporation with no significant difference in results between these two solvents. Their use probably counteracts the adverse capillarity phenomenon because of their low surface tension and evaporation speed.

The observation of this macroscopic change in shape of specialized scales sheds a new light on the cause of the colour change for *Phorinia* specimens. Hypotheses previously proposed by entomologists to explain this fact such as exudation of body fats or oxidation of pigments (C. Raper, pers. comm.) are invalidated by our observations. The change of macroscopical conformation of scales, which also affects the microscopical level, resulting in a dull yellow colour lead us to consider the hypothesis that the vivid bluish-green colour observed in living (or alcohol preserved) specimens results from the combination of a yellow pigment with a photonic crystal microstructure, present in the cuticule of these scales, which reflect a blue light. Indeed the combination of yellow and blue light produce a blue-green colour. Further studies are currently underway to elucidate the exact mechanisms in the scales of *Phorinia* spp.

The combination of structural and pigment coloration to produce a new colour is found in birds and also in many other animals, for example butterflies, beetles and lizards (Kinoshita 2008). It has recently been observed in the orange feathers of the common kingfisher, *Alcedo atthis* (Linnaeus 1758) or the green feathers of several Neotropical parrots (Stavenga et al. 2011, Tinbergen et al. 2013). In the second case, the vivid green colour of barbs results from a combination of spongy nanostructured barb cells partly enveloped by a blue-absorbing, yellow-colouring psittacofulvin pigment. The nanostructure reflects the blue or blue-green wavelength range and the pigment acts as a spectral filter for yellow. This kind of combination results in bright and saturated colours.

The roles of these specialized scales in *Phorinia* and other taxa of the Tachinidae are of interest. It has been demonstrated that for birds, butterflies, damselflies and beetles, vivid and iridescent colours play a major role in mating and partner choice (Fitzstephens and Getty 2000, Kemp 2007, Kuitunen and Gorb 2011, Kemp et al. 2014). They may reveal an individual’s stress and health (McGraw et al. 2002, Meadows et al. 2012) or may be used to enhance camouflage by matching the reflectance of the surrounding background as for *E. imperialis* (Curculionidae) or *Prosopocera lactator* (Fabricius, 1801)(Cerambycidae) (Colomer et al. 2012, Wilts et al. 2012). Recently, Cerretti et al. (2014b) studied cuticle areas in the Tachinidae which exhibit specialized exocrine glands and hairs. The specialized scales of *Phorinia* spp. do not currently appear to be linked to an exocrine function but they may undertake other roles. Further field data will help our understanding. Moreover, the existence of such scales and colouration in several other genera of Tachinidae should be investigated from a phylogenetic point of view (examination of scales for one undetermined species of *Chrysoexorista* previously kept in alcohol show a different shape). They may provide new taxonomic information in a similar approach to that obtained for species of the *Tychius* genus (Coleoptera: Curculionidae) (Erbey and Candan 2013).

Our tests have demonstrated the effectiveness of methods to preserve the original vivid colours on Ethanol dehydrated specimens of Tachinidae, some of which are simple and effective. We draw the attention of field collectors to the fact that the vivid blue-green colour of field specimens will disappear if they are air dried rather than immersed in alcohol and subsequently treated as suggested above. Moreover our study provides evidence for the existence of a mechanism for colour production linked to specialized scales that has previously not been reported in Tachinidae. These structures and the colour production mechanism raise new phylogenetic and ecological questions such as the rule of such scales and colours in several genera of the same subfamily and the evolutionary processes to acquire such characters.

## Supplementary Material

Supplementary material 1 *Phorinia* sp. from Zambia, air dried Data type: images
Brief description: Photo of a undetermined *Phorinia* sp collected in Zambia. Dorsal view of the tomentose area which were green in live and fade to a yellow colour once air dried.
File: oo_59587.jpg Stephen Downes

## Figures and Tables

**Figure 1a.**
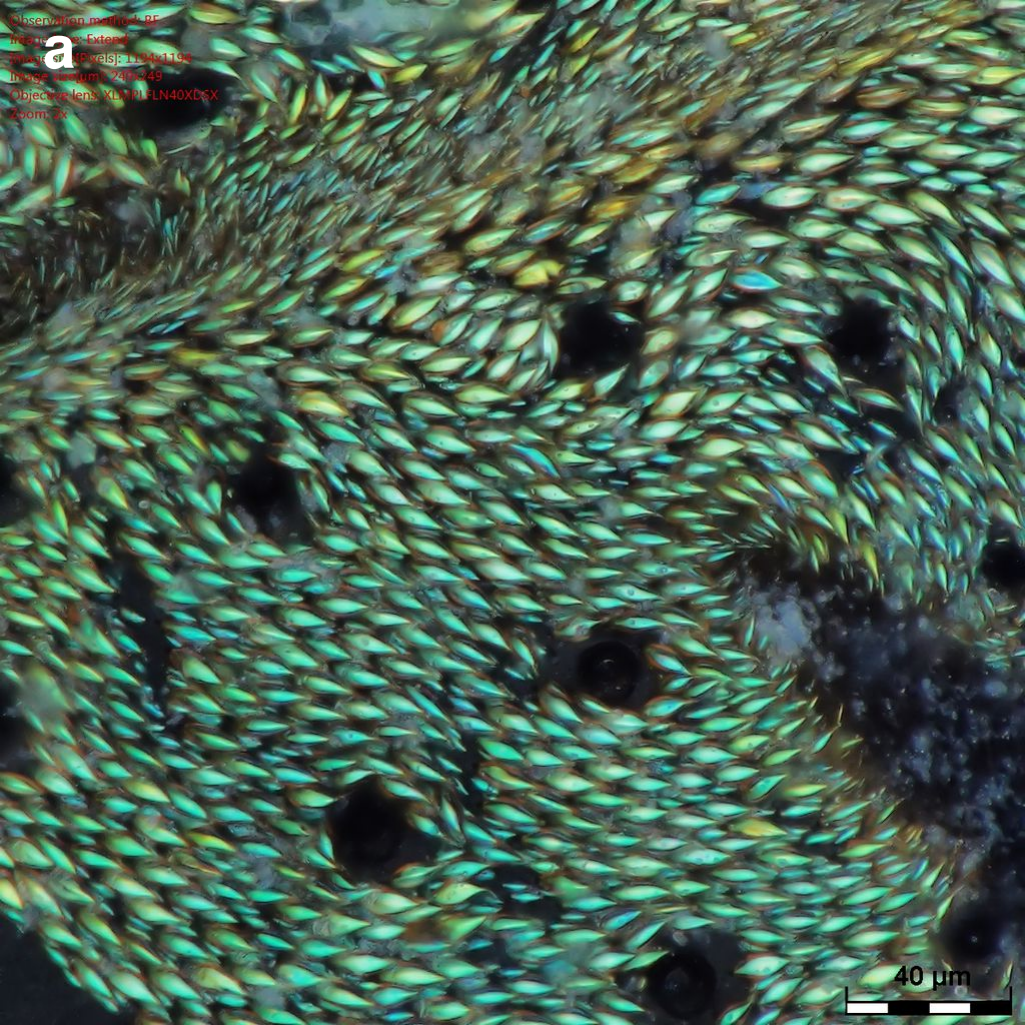
Scales dried by the critical drying point method under optical microscope.

**Figure 1b.**
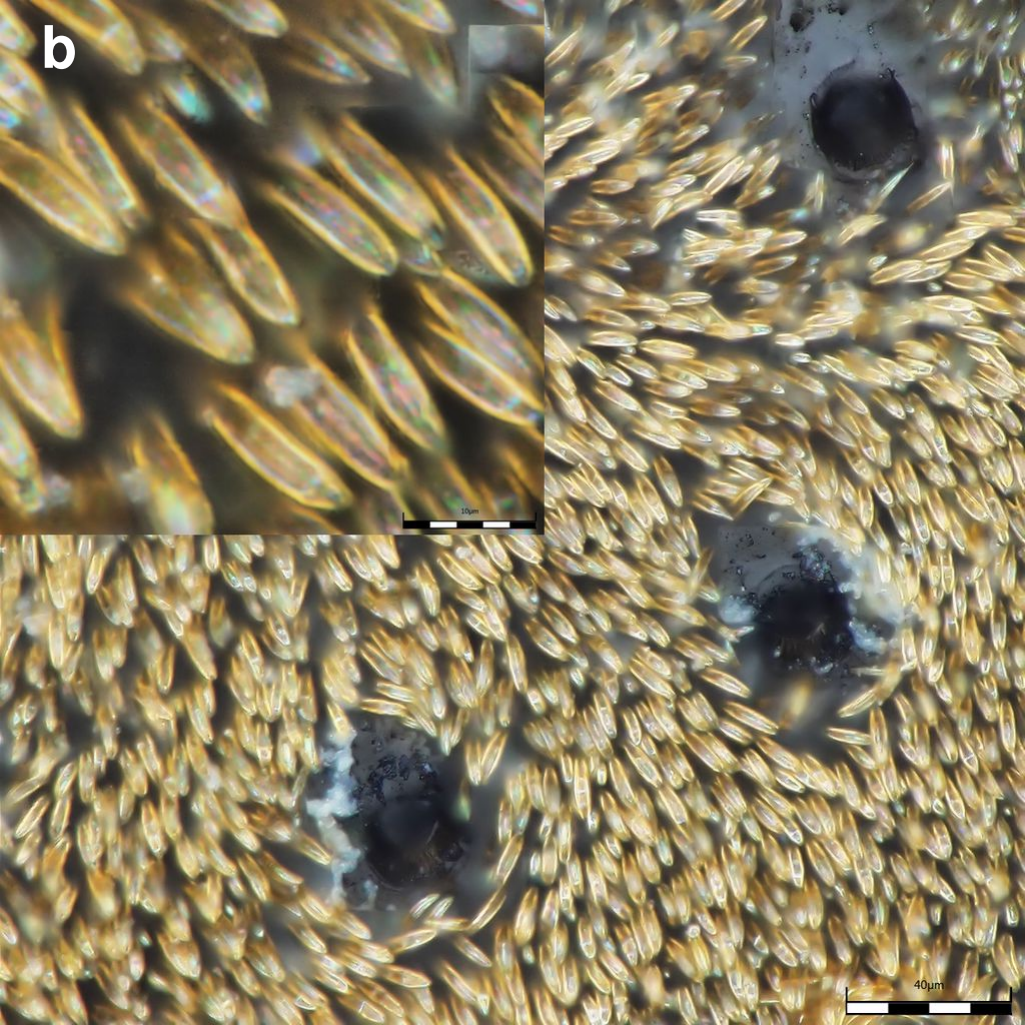
Scales dried in open air at room temperature under optical microscope.

**Figure 2a.**
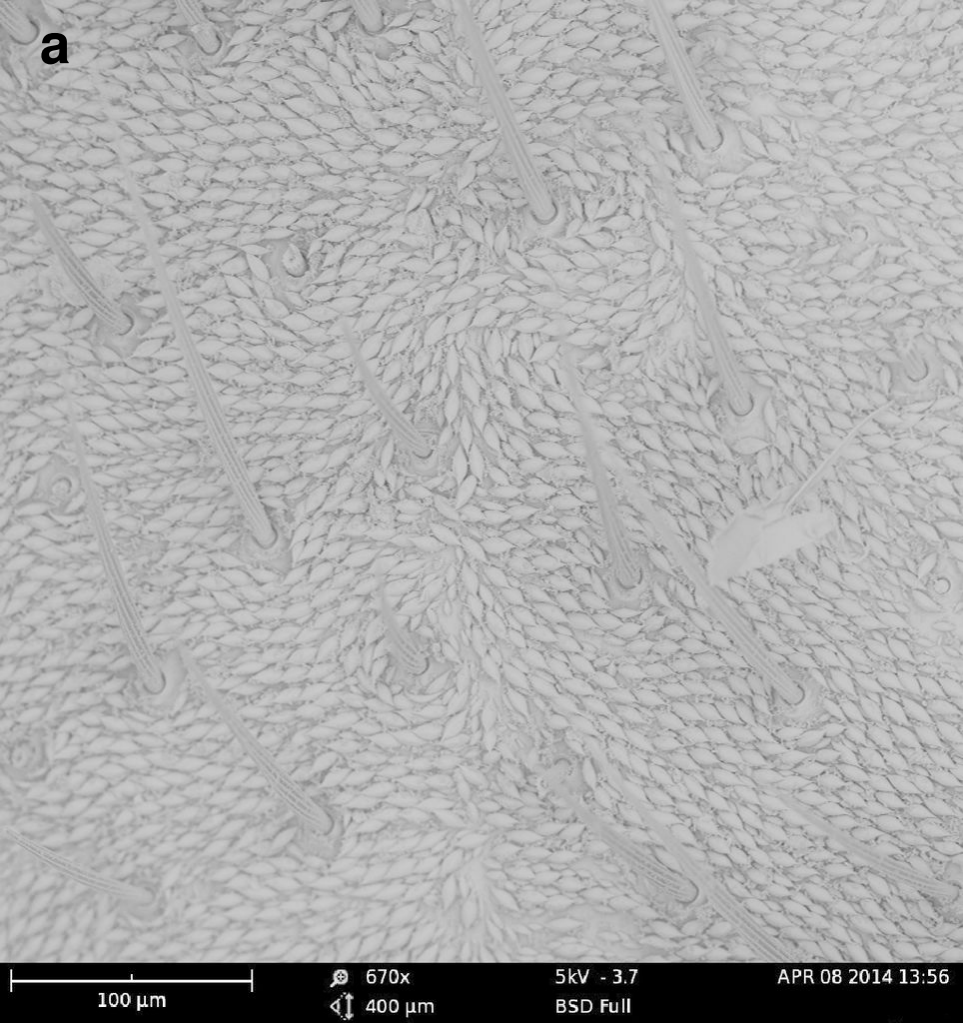
Scales dried by the critical drying point method under SEM.

**Figure 2b.**
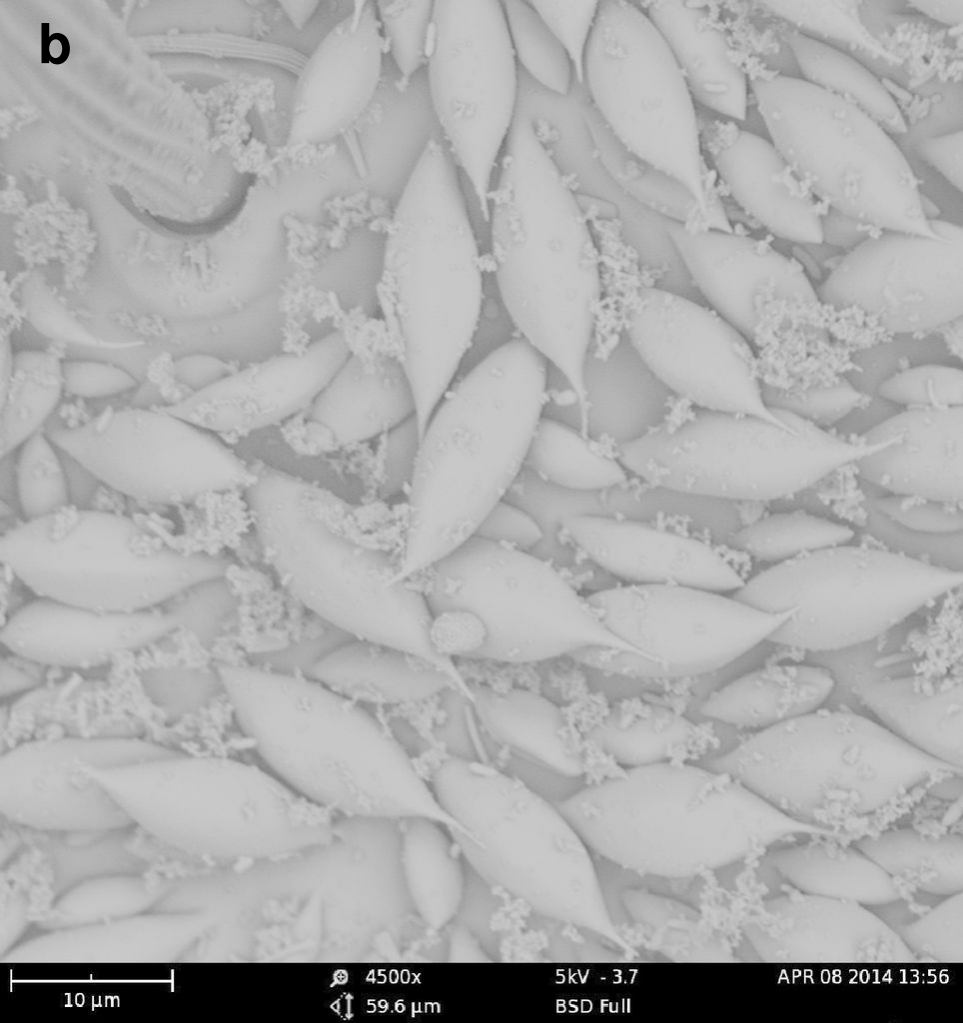
Scales dried by the critical drying point method under SEM - detail.

**Figure 2c.**
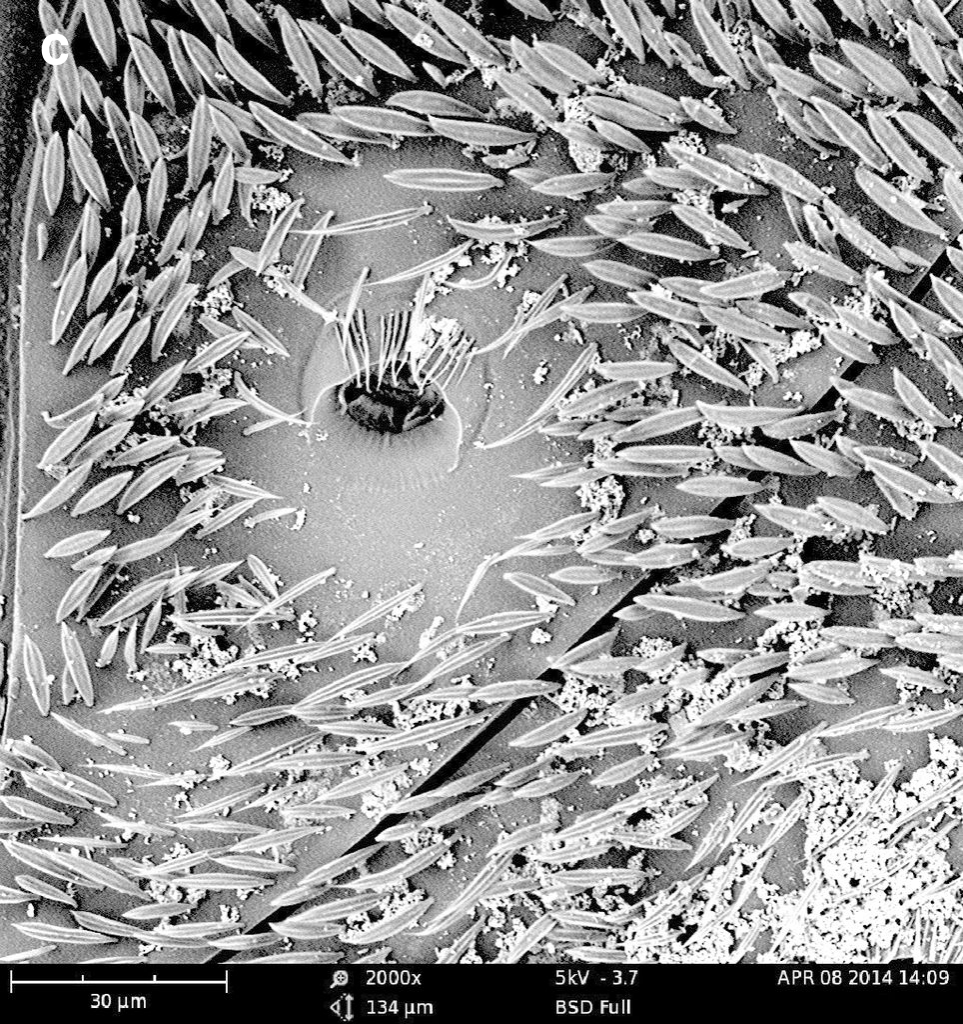
Scales dried in open air at room temperature under SEM.

**Figure 2d.**
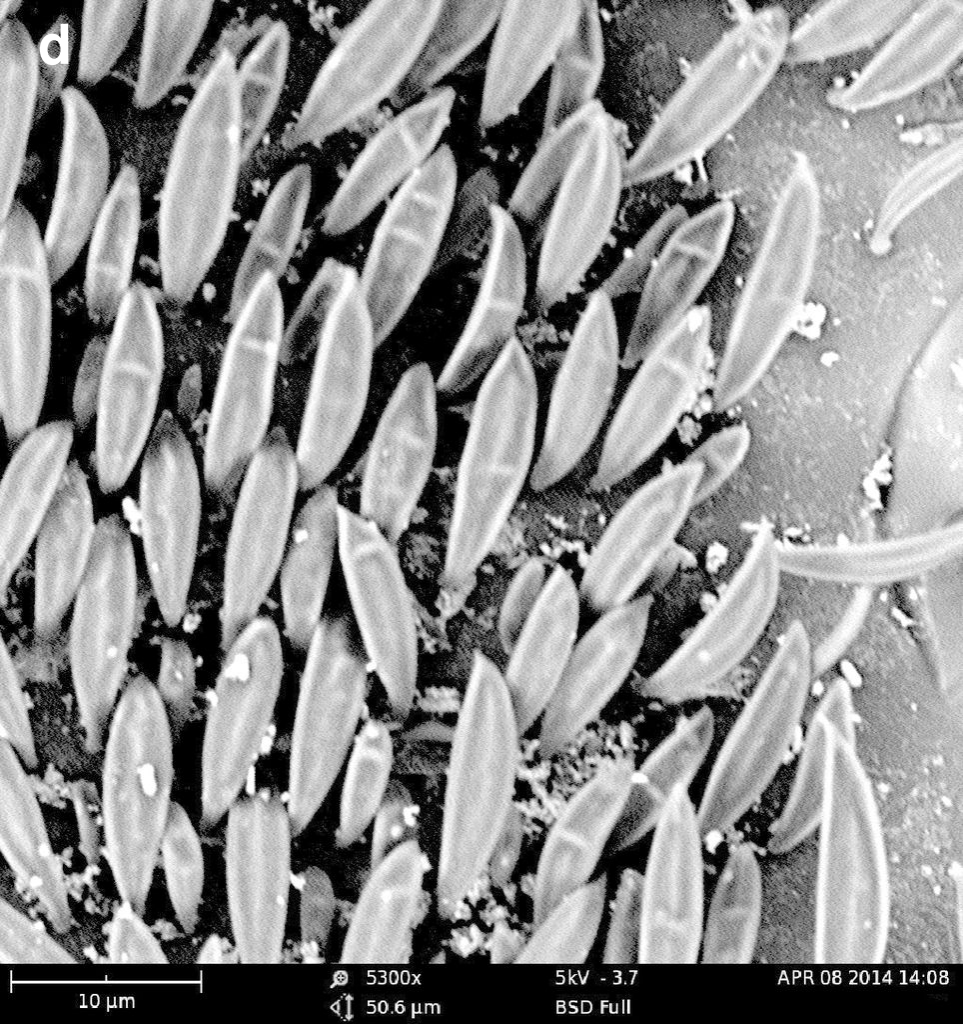
Scales dried in open air at room temperature under SEM - detail.
