# Supplementary figures and images for: Preservation of iridescent colours in Phorinia Robineau-Desvoidy, 1830 (Diptera: Tachinidae)
Source: Biodivers Data J. 2016 Jan 7;(4):e5407. doi: 10.3897/BDJ.4.e5407 (PMC4759442; doi:10.3897/BDJ.4.e5407)

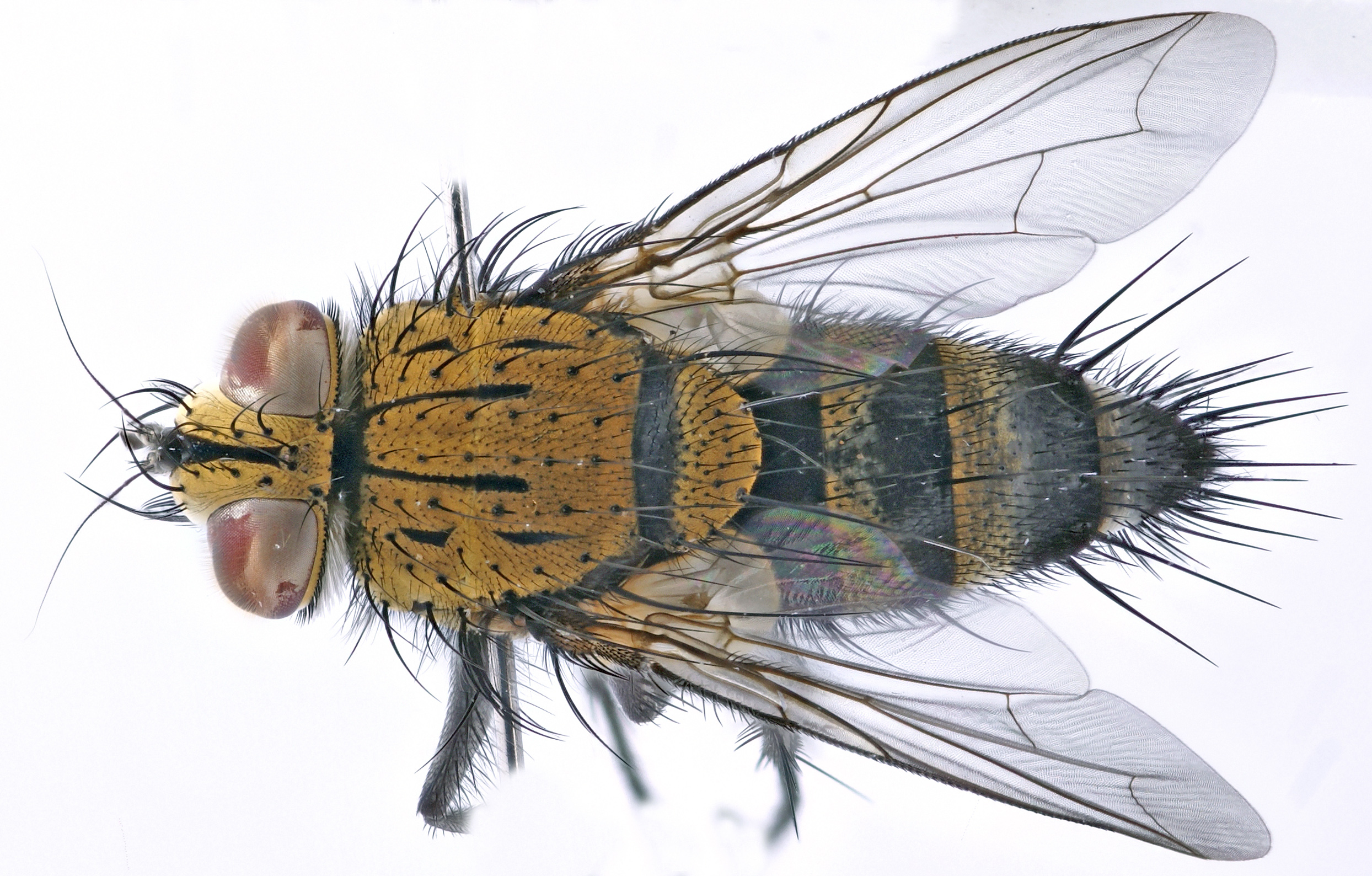

Supplement: Supplementary material 1 — Phorinia sp. from Zambia, air dried [file biodiversity_data_journal-4-e5407-s001.jpg]
